# Contribution of oxidative stress in the mechanisms of postoperative complications and multiple organ dysfunction syndrome

**DOI:** 10.1080/13510002.2021.1891808

**Published:** 2021-02-23

**Authors:** Javier Toro-Pérez, Ramón Rodrigo

**Affiliations:** a Faculty of Medicine, University of Chile, Santiago, Chile; b Molecular and Clinical Pharmacology Program, Faculty of Medicine, Institute of Biomedical Sciences, University of Chile, Santiago, Chile

**Keywords:** Surgery, oxidative stress, organ failure, sepsis, antioxidants

## Abstract

**Background:**

The extent of the damage following surgery has been subject of study for several years. Numerous surgical complications can impact postoperative quality of life of patients and even can cause mortality. Although these complications are generally due to multifactorial mechanisms, oxidative stress plays a key pathophysiological role. Moreover, oxidative stress could be an unavoidable effect derived even from the surgical procedure itself.

**Methods:**

A systematic review was performed following an electronic search of Pubmed and ScienceDirect databases. Keywords such as sepsis, oxidative stress, organ dysfunction, antioxidants, outcomes in postoperative complications, among others, were used. Review articles were preferably used between the years 2015 onwards, not excluding older ones.

**Results:**

The vast majority point to the role of oxidative stress in generating greater damage and worse prognosis in postoperative patients without the necessary care and precautions, taking importance on the use of antioxidants to prevent this problem.

**Discussions:**

Oxidative stress represents a common final pathway related to pathological processes such as inflammation or ischemia–reperfusion, among others. The expression of greater severity of these complications can result in multiple organ dysfunction or sepsis. The aim of this study was to present an update of the role of oxidative stress on surgical postoperative complications.

## Introduction

Since its inception, surgery has been considered a branch of medicine characterized by the postoperative trauma that it can generate in the patient. Thus, during recent years, there has been a growing interest not only in reducing the adverse effects of surgery but also in preventing the appearance of some secondary pathologies and thus improving the quality of life of patients. The achievement of these aims should contribute to reduce the cost-burden in health systems caused by longer periods of hospitalization and subsequent corrective procedures. Each surgery can induce a diverse extent of physiological stress in the body, which could lead to the development of postoperative complications. For example, the body response might involve the stimulation of endocrine changes, generating possible metabolic sequelae [1], among other consequences.

Common postoperative complications include sepsis, organs diseases, fever, hemorrhage, deep vein thrombosis, among others. These complications have rates as high as 30%. It has been developed increasing effort to apply some surgical programs aimed to improve the clinical outcome of patients [2]. Readmissions after an acute care hospitalization or surgery are frequent, affecting an average of 18% of patients within 30 days after hospital discharge. These complications manifest as an expensive cost for the healthcare systems and for the patients [3]. It was reported that within 6 months following discharge, approximately 32% of surgical patients had an unplanned readmission and 10% died [4]. One of the greatest challenges of any physician is to successfully face these complications by preventing, detecting, and managing these issues, for the reason of the high mortality that can be exposed to these patients after a surgical procedure [5]. The pathogenesis of these complications is multifactorial, but one focus for clinicians includes the occurrence of oxidative stress. The relevance of oxidative stress appears as a common pathway within many possible complications following surgery. The aim of this review is to present a contemporary report explaining some effects accounting for the role of oxidative stress in the pathophysiology of the most common postoperative complications and the implications of identifying these pathways for the development of potential therapeutic agents or revised clinical approaches that provide benefit in improving the clinical outcome of patients.

## Oxidative stress

Oxidative stress can be defined as a significant uncontrolled generation of reactive oxygen species (ROS) and reactive nitrogen species (RNS) that overwhelm the activity of the endogenous antioxidant system [6]. Cellular biomolecules that are primarily aﬀected by ROS and RNS are proteins, lipids, and DNA [7]. Physiologically small amounts of ROS are produced in the cells. For example, ROS are mediators in complex cellular processes and signaling networks, such as prostaglandin production pathways, mitochondrial respiration and host defense [8]. Regardless of the mechanism associated with cell signaling, ROS are in charge of regulating the activities of antioxidant enzymes in cells. For example, we have the case of Nrf2 (nuclear factor erythroid 2 related factor 2), which is a transcription factor that up-regulates the expression of several antioxidant and detoxifying genes, all thanks to its ability to bind to promoter sequences that contain a consensus antioxidant response element [9]. Furthermore, ROS not only participate in the regulation of antioxidant gene expression but are also capable of interacting with cell signaling molecules such as MAP kinases, PI3 kinase, and protein tyrosine phosphatases. All of this leads to cascades of signaling involved in various cellular processes, including proliferation and survival [10]. However, and as stated above, when this system is deregulated it can cause important cellular and pathophysiological changes that will be explained below.

### Prooxidants effects

ROS are a family of highly reactive species that are formed either from enzymatic and non-enzymatic sources. Enzymatic sources include the xanthine oxidase system, NADPH oxidase system, mitochondrial electron transport chain and uncoupled nitric oxide synthase (NOS) system. Reactive oxygen species are mainly free radicals, such as superoxide radical anion (O_2_^·^−) and hydroxyl free radical (HO^·^), since they have unpaired ‘reactive’ electrons in their molecule. Among these, the HO^·^ stands out, as it is considered the most powerful and aggressive oxidant, mainly responsible for the oxidative damage of the DNA bases. In addition, other reactive oxygen species, such as hydrogen peroxide (H_2_O_2_), hypochlorous acid (HOCl) and singlet oxygen (^1^O_2_) molecules, are formerly not free radical molecules but are classified as reactive oxygen species [11]. One characteristic of H_2_O_2_ is that it can form HO^·^ through the Fenton reaction in the presence of free iron, thus causing lipid peroxidation and DNA damage [12]. In turn, reactive nitrogen species include the free radical nitric oxide (NO^·^) and the nonradical peroxynitrite anion (ONOO−) The production of NO^·^ and ONOO − occurs via the endothelial nitric oxide synthase (eNOS). Normally eNOS synthesizes nitric oxide through the transfer of electrons from tetrahydrobiopterin (BH_4_) to L-arginine, producing L-citrulline and NO in an enzymic process that requires oxygen and NADPH. At low levels of BH_4_, eNOS is uncoupled by passing the L-arginine electrons to oxygen, thus generating O_2_^·^− and then the nonradical potent 2-electron oxidant, peroxynitrite anion, which in turn decreases the levels of BH_4_ [13] in a cycle that leads to enhanced production of peroxynitrite anion.

### Antioxidants effects

The oxidative stress defense system consists of antioxidant enzymes and molecules with the ability to donate electrons, the latter called antioxidants. Antioxidants are nucleophilic and reducing molecules, which have the ability to react with oxidants such as ROS, reducing them to a less reactive state. The main antioxidant enzymes in the body are superoxide dismutase (SOD) which dismutates superoxide anion to less reactive hydrogen peroxide, catalase (CAT) which also catalyzes the breakdown of H_2_O_2_, and glutathione peroxidase (GSH-Px) which catalyzes the conversion of H_2_O_2_ into water. Glutathione peroxidase oxidizes a low molecular weight substance called reduced glutathione (GSH) into oxidized glutathione (GSSG). GSH is one of the most important redox buffers for the cells, since it can be found in all cell compartments, and can react in several redox enzymatic processes [14]. Another antioxidant defense mechanism is the oxidation of mitochondrial thioredoxin-2 (TRX-2), along with its associated enzymes such as peroxiredoxin-3 and thioredoxin reductase-2. It has been seen that in sepsis conditions, where there is a severe oxidative environment, the TRX-2 system takes on a greater role, because it has been seen that proteins of the TRX-2 system were more resistant to the effects of oxidative stress and have a more important role in protecting against mitochondrial dysfunction than the GSH system, under conditions of sepsis in human endothelial cells in culture [15].

## Impairment of redox balance by surgery

**Figure 1.**
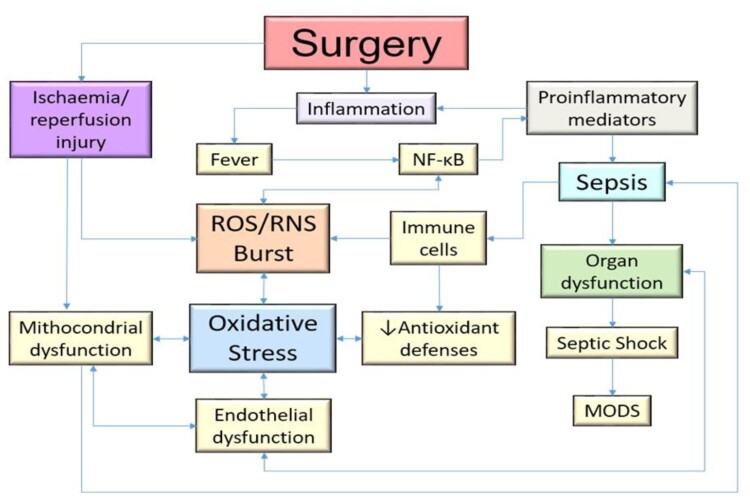
A panoramic vision of the role of oxidative stress in pathophysiological mechanism in the body. Surgery can be the main starter in all this process. MODS, Multiple Organ Dysfunction Syndrome; ROS, Reactive oxygen species; RNS, Reactive nitrogen species; NF-ĸB, Nuclear Factor kappaB.

Surgery is an acute event that causes significant inflammation and ischemia/reperfusion injury (IRI), and ROS play an important role as mediators of this damage. The magnitude and the technique of the surgical intervention have a huge influence on the intraoperative oxidative stress response. In the most complex surgeries, higher levels of oxidative stress have been found compared to less invasive counterparts [16] (Figure 1).

## Inflammation

Inflammation is a complex, conserved and non-specific biological response of cells to injurious damage. During surgery, it is logical that tissue damage occurs, so specialized cells such as macrophages and neutrophils secrete inflammatory mediators called cytokines, producing local inflammation, which can become a systemic inflammation in severe cases, and prostaglandins, produced by non-immune cells of the injured tissue [17].

Generally, the inflammatory response is initiated by the activation of pattern recognition receptors, such as Toll-like receptors (TLRs) and NOD-like receptors (NLRs), which are expressed on the surface of neutrophils and macrophages. Activation of these receptors occurs after the recognition of pathogen-associated molecular pattern molecules (PAMP) and damage-associated molecular pattern molecules (DAMP), causing changes in inflammatory cell signaling pathways [18]. In the case of TLRs, these induce intracellular cascades, activating downstream kinases such as IkB and MAP kinases that regulate different transcription factors such as NF-κB and AP-1, which finally induce the expression of pro-inflammatory genes and the synthesis of associated proteins to inflammation, such as cytokines (IL-6, TNF-α, IL-1β) and chemokines. Cytokines can change vascular permeability and endothelial function by increasing adhesion molecules such as L-selectin and E-selectin, causing rolling along the vascular endothelium, with the consequent activation of integrins. These molecules bind to vascular endothelial adhesion molecules, such as ICAM-1 and VCAM-1, facilitating the transmigration of leukocytes through the activated endothelium to the site of injury [19]. The utility of chemokines lies in recruiting additional immune cells and proteins such as iNOS that generate nitric oxide (NO) [20]. Instead, NLRs are involved in the formation of multiple protein signaling complexes known as inflammasomes. The inflammasome is composed by a nucleotide-binding oligomerization domain (NOD), which in turn contains domain 3 of pyrin (NLRP3). Hence the importance of the inflammasome because it is necessary for the intracellular processing of pro-IL-1β and the secretion of active IL-1β, both of the cytokine family [21]. Finally, high levels of reactive oxygen species (ROS) have been shown to activate NLRP3 [22].

Another important aspect of ROS to consider is that phagocytes have the ability to synthesize these compounds, because they have oxidant-generating enzymatic systems, such as the macrophage and its membrane-bound NADPH oxidase system. This produces O_2_ and myeloperoxidase, which later converts H_2_O_2_ and Cl into HOCl and ^·^OH [23]. ROS have another role for macrophages, but some details should be explained first. Macrophages can be divided into subtypes M1 and M2. Broadly speaking, M1s have been associated with microbicidal and pro-inflammatory functions, while M2s with cell proliferation and wound healing. These functions are explained by the fact that M1 responds to TLR agonists and IFN-γ produced by Th1 lymphocytic cells, which activate iNOS to produce NO and ROS to kill pathogens and secrete pro-inflammatory cytokines (IL-8, TNF-α and IL-1β), especially IL-12 which improves Th1 responses [24]. Relating what has been described to oxidative stress, it has been shown that ROS are capable of increasing the synthesis of pro-inflammatory cytokines, which increases the M1 phenotype and with it the damage and inflammatory action in an unregulated way [25]. Likewise, the MAPK family, such as p38, c-Jun NH2-terminal kinase (JNK) and extracellular signal-regulated kinase (ERK), are phosphorylated, leading to the expression of pro-inflammatory mediators in activated macrophages [26]. Furthermore, it has been seen that NF-kB is also capable of acting by increasing the M1 phenotype. Finally, ROS are related to the activation of the inflammasome and its dysregulation in the M1 phenotype [27].

## Ischemia/reperfusion injury

Ischemia–reperfusion injury (IRI) is a critical condition that always appears in more complex surgeries, so its knowledge is mandatory to control cell damage and preserve organ function. IRI is classically associated with interventions such as organ transplantations, the use of limb tourniquets and vascular clamps [28]. In addition, associated with IRI, hypoxia has an important role in the hypoperfusion of tissues, resulting in an excessive production of ROS leading to oxidative damage [29]. Pathophysiologically, in ischemia there is a dysfunction of the Na^+^-K^+^-ATPase pump, which produces an overload of sodium and calcium in the cytoplasm. This accumulation of calcium causes the activation of multiple cytoplasmic proteases responsible for degrading proteins, as well as activating apoptotic processes in the mitochondria [13]. Also in this process, without oxygen, anaerobic glycolysis and mitochondrial dysfunction are enhanced, leading to reduced intracellular pH; whereas reperfusion gives rise to excessive ROS production due to increased oxygen delivery [30]. In reperfusion damage, this generates a significant electrolyte imbalance, with the systems already described playing an important role. In particular, the xanthine oxidase system has a point in common with these redox changes. At first, ATP undergoes enzymatic processes leading it to convert into hypoxanthine. This system has the function of producing ROS by oxidizing hypoxanthine to xanthine and xanthine to uric acid. Under low O_2_ conditions, such as ischemia, the enzyme xanthine dehydrogenase displaces xanthine oxidoreductase due to lower levels of ATP, which induces the formation of ROS during the conversion of hypoxanthine to uric acid, which ultimately further aggravates the oxidative environment [28]. In the case of hypoxia, this condition induces the functions of hypoxia inhibitory factor-1α (HIF-1α) to be activated, which act, for example, to promote the activation of NOX enzymes, which is increasing even more for oxidative stress. Consequently, after the restoration of blood flow, different mediators are released (phospholipase A2, TNF-α, IL1β, IFN-y, among others) by cells, which are capable of increasing the action of NADPH oxidase [31]. Many cells are able to attract nearby neutrophils amplifying the response to inflammatory mediators. Neutrophils are sensitized or primed to activating factors, such as chemotactic cytokines, therefore, generate much larger amounts of ROS which explains the late ROS generation during reperfusion [32] and during the earliest stages of postoperative recovery.

## Common postoperative complications associated with oxidative stress

### Fever

One of the most common complications in patients after a major surgery is the postoperative fever, defined as an elevation of body temperature to greater than 38.3°C. Many studies indicate a range incidence between 14 and 91% of postoperative fever in many surgical subspecialties [33]. As a normal response for an acute event like a surgery, macrophages and monocytes secrete specific pyrogenic and inflammatory cytokines: IL-1, IL-6, TNF-α and interferon-gamma [34]. Within the pathophysiology of fever, it is related to the immune and inflammatory mechanisms already described. IL-6 contributes to the COX-2 PGE2 pathway. Fever has typically been classified into two phases: an early and a late one. During the early phase, it is postulated that the generation of cytokines is due to PGE2 synthesized in the macrophages of the lungs and liver. In contrast, the late phase of fever is mediated by PGE2 produced at the level of the blood–brain barrier, by endothelial cells or perivascular macrophages of the preoptic hypothalamus. For this reason, IL-6 can be an important inducer of COX-2 in the later stages of fever, although recent studies have shown that IL-6 can act through mechanisms independent of COX-2 [35]. It has been shown a directly correlated magnitude of these substances and the fever curve in first days of postoperative period [36].

### Relation between sepsis and oxidative stress

A severe complication after surgery is sepsis, which began with an inﬂammatory responses associated with oxidative stress [37]. Loss of integrity of the external barrier allows the development of an inflammatory response through the release of pro and anti-inflammatory mediators. Also, during sepsis, endothelial cell damage leads to the loss of microvascular barrier function, resulting in enhanced vascular permeability, which leads to activated neutrophil and platelet adhesion. It has been demonstrated an improvement of inflammatory mediators NF-ĸB, cytokines and pentraxin-3 levels in patients with sepsis [15]. In different conditions, leukocytes can secrete hydroxyl radical, nitric oxide metabolites, O_2_^·^− and other ROS/RNS species [38]. An enhancement of inducible nitric oxide synthase (iNOS) activity is caused by NF-ĸB activation and lipopolysaccharide (LPS) treatment, producing higher concentrations of NO, which can be combined with superoxide anions to form ONOO− [39]. Likewise, imbalances in the SOD: CAT ratio have been associated with increased oxidative stress and morbidity in sepsis. Superoxide is usually converted to H_2_O_2_ by manganese-containing SOD (Mn-SOD) and then to H_2_O by CAT. However, under conditions of high oxidative stress such as sepsis, the concentrations of these enzymes can change and this may lead to an accumulation of damaging ROS [40].

Sepsis is also associated with mitochondrial dysfunction as a consequence of oxidative stress, where NO and ROS combined with the variety of inflammatory mediators have direct or indirect influence on the energy production and mitochondrial function [41]. Specifically, the peroxidation of the mitochondrial lipid cardiolipin enhances the ROS production by the dissociation of cytochrome c, which induces low ATP levels [42]. With all the above, an oxidant environment can produce changes in proteins, lipids and DNA structure, leading to lack of ATP which produces necrosis, increased vascular permeability by damage in endothelial cells and, after all, a worsening of sepsis [43]. In different studies, cellular energy failure is demonstrated in sepsis cases. This is associated with worse outcomes in critically ill patients, especially due to mitochondrial dysfunction [38,39,44]. With regard to the pathophysiology of mitochondrial dysfunction, there is a depletion of intracellular ATP levels, which triggers alteration of Ca^2+^ homeostasis, excessive production of ROS and release of pro-apoptotic proteins. All this, at the same time, is associated with the transition of mitochondrial permeability, because an association has been seen between inflammation of the matrix and the uncoupling of the respiratory chain. Likewise, other intramitochondrial processes occur such as Ca^2+^ outflow, loss of membrane potential, increased ROS synthesis, and release of cytochrome c from mitochondrial complexes, leading to apoptosis [45]. Finally, damage to the mitochondrial membrane can lead to the activation of the caspase pathway, with the consequent release of cytochrome c and apoptosis [15]. The NADPH family of oxidases also plays an important role in the development of sepsis and oxidative stress. So far, seven isoforms of Nox (NOX1, NOX2, NOX3, NOX4, and NOX5; Duox1 and Duox2) have been identified, all with distinct catalytic domains. Among these, NOX4 stands out, because it has been seen that different inflammatory stimuli (LPS in Gram negative bacteria, TNF-α, TGF-β and hypoxia) can activate NOX4 and this generates a greater amount of ROS. Other subtypes with their respective tissue dysfunction will be detailed later [46].

In the worst of cases, sepsis can develop into septic shock, which is accompanied with hypotension that persists after resuscitation with intravenous fluid or hyperlactatemia [47]. Several factors such as hypotension and microvascular thrombosis, contribute to decreased oxygen levels, and in addition with mitochondrial damage caused by oxidative stress and other mechanisms like inflammation, generates a vicious cycle that enhance oxidative stress that exacerbates the pathophysiology of septic shock [48].

### Platelets dysfunction

Platelets play an important role in septic patients. High levels of platelet ROS have been shown to be associated with different thrombotic pathologies, such as hypertension, diabetes, and metabolic syndrome. Likewise, ROS are capable of interacting in different pathways of platelet aggregation, being able to cause a dysregulation in coagulation [49]. Structurally, platelets possess a transmembrane receptor that is a member of the immunoglobulin (Ig)-like superfamily called GPVI [50]. Under normal conditions, platelets basally contain intracellular ROS, which increase after stimulation of the GPVI receptor. Currently, the only proven evidence of the role of platelets in oxidative stress has been the binding between GPVI and tumor necrosis factor receptor (TRAF4). The action of TRAF4 within this association is that it binds selectively to the cytoplasmic tail of GPVI and interacts with a subunit of the NADPH oxidase 1 and 2 complex (NOX1/2) called p47phox. This NOX1/2 complex is characterized as being the main source of ROS production in platelets [51]. The use of compounds such as diphenylene iodonium chloride (DPI) and apocinin, which act as inhibitors of NOX activity, significantly impeded the activation, aggregation and formation of platelet thrombi [52].

### Endothelial dysfunction

**Figure 2.**
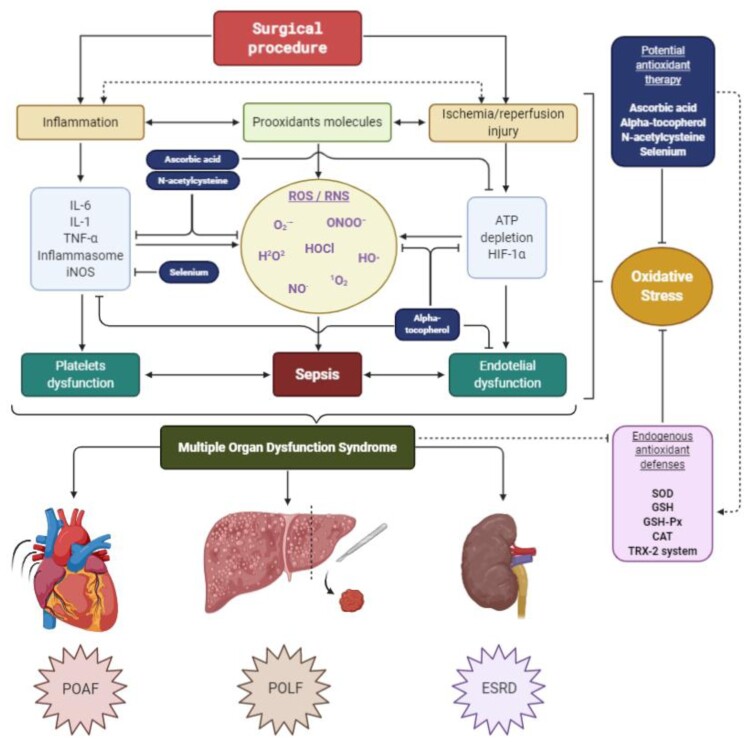
Relationship between cytokines and platelet and endothelial dysfunction in the generation of sepsis and subsequent MODS. Likewise, the loss of energy and the non-degradation of HIF-α can contribute to increase the damage in the mitochondrial enzymes of the oxidative system, being able to cause an increase in the generation of ROS. The antioxidant therapy described has multiple targets. Likewise, it has direct and indirect antioxidant activity, increasing the levels of the endogenous antioxidant system. MODS, Multiple Organ Dysfunction Syndrome; ROS, Reactive oxygen species; RNS, Reactive nitrogen species; O_2_^·^−, Superoxide radical anion; HO^·^, Hydroxyl free radical; H_2_O_2_, Hydrogen peroxide; HOCl, Hypochlorous acid; ^1^O_2_, Singlet oxygen; NO^·^, Free radical nitric oxide; ONOO−, nonradical peroxynitrite anion; IL-6, Interleukin 6; TNF-α, Tumor necrosis factor alpha; IL-1, Interleukin 1; iNOS, Inducible nitric oxide synthase; ATP, Adenosine triphosphate; HIF-1α, Hypoxia-inducible factor 1-alpha; SOD, Superoxide dismutase; GSH, Reduced glutathione/GHS-Px, glutathione peroxidase; CAT, Catalase; TRX-2, Mitochondrial thioredoxin-2; POAF, Postoperative atrial fibrillation; POLF, Postoperative liver failure; ESRD, End-stage renal disease.

Endothelial dysfunction has been described as one of the main mechanisms of morbidity and prognosis in septic patients and is defined as an imbalance in the production of the vasodilator and vasoconstrictor factors [53]. The endothelium is closely related to vasodilation, vasoconstriction, platelet adhesiveness, leukocyte transmigration, inflammatory and immune response, among many other functions. The endothelium secretes a variety of molecules: NO and prostacyclin (PGI2) have vasodilator, antithrombotic and antiproliferative properties; ET-1 and AngII, with vasoconstrictor effects; von Willebrand factor (vWF) and plasminogen activator inhibitor-1 (PAI-1), with prothrombotic functions; and anticoagulants, such as tissue plasminogen activator (tPA) [54]. Among all the molecules already mentioned, NO is outlined as one of the most important in endothelial dysfunction, since low levels of it cause this imbalance [55]. High levels of ROS directly attack endothelial cells and displace the action of oxidative systems, which causes a decrease in the ability of iNOS to generate NO and leading to a pro-inflammatory state. This leads to an increase in vascular permeability, which is classically observed in septic patients with hypotension. Likewise, another molecule related to this dysregulation of NO is the overexpression of NOX4 in the endothelium, because it has been shown to improve vasodilation and reduce blood pressure, through a greater production of H_2_O_2_ and a reduction in the inactivation of NO [56]. On the other hand, ROS cause an imbalance in oxygen consumption, which, together with hypoxemia in these types of patients, accelerates the systemic deterioration of the organs, leading to generalized ischemia, decreased perfusion and finally to multiple organ failure [44] (Figure 2).

### Potential biomarkers in sepsis

The knowledge in recent years of new biomarkers in sepsis has been crucial for the diagnosis and prognosis of this entity. In general, a biomarker is any component of natural origin through which physiological or pathophysiological processes can be recognized [57]. For a biomarker to be efficient and of maximum utility, it must be able to establish an early diagnosis, stratify the severity and risk, and be able to monitor the treatment of the disease [58]. Classically in sepsis, the most widely used biomarkers lack adequate specificity and sensitivity for diagnosis and prognosis, if used individually. The traditional and most commonly used in the clinic are inflammatory biomarkers such as CRP, IL-6, lactate and procalcitonin (PCT), because an increase in these compounds has been seen in septic conditions [59]. In the first one, C-reactive protein (CRP) is an acute phase reactant stimulated by the action of IL-6 in hepatocytes in inflammatory phenomena or tissue injury. It is commonly used as a marker of inflammation and infection [58]. One of the defects of this biomarker is that by itself it is a poor predictor of mortality and specificity compared to other biomarkers, so it must be used in conjunction with others to make a diagnosis or prognosis with more certainty [60]. Another widely used biomarker is lactate, which can more accurately represent organ dysfunction. It has been shown that patients with hyperlactatemia (elevated serum lactate levels in the blood) are associated with higher rates of mortality and morbidity in the future, because this biomarker, which is closely related to the anaerobic metabolism of pyruvate, is able to reflect tissue hypoperfusion in sepsis [59,60]. Even so, the most widely used and accepted biomarker in different clinical guidelines in sepsis is procalcitonin (PCT), because it has shown better specificity and sensitivity than the other biomarkers already mentioned, as well as more efficiently reflecting the patient's inflammatory parameters [61].

Among the oxidative markers used clinically, are preferred those capable of measuring the stable end products of ROS damage to different components of cells, such as membrane lipids, cellular proteins and nuclear constituents. Some examples are malondialdehyde (MDA), F2-isoprostanes, 8-hydroxy-2'-deoxyguanosine, and many more that will not be explored in detail in this review. Other useful markers to measure are the endogenous antioxidant enzymes already described, such as GPx, CAT, and SOD. Also the reduced / oxidized thiol ratio has served as a marker. The difficulty of using these enzymes lies in the low specificity they have in an organ and, likewise, their general interpretation within the oxidative state of a septic patient [62]. At present, the field of research for new biomarkers within sepsis has grown enormously. There are new technologies capable of generating an electrochemical reading composed of the general redox potential (ORP), biological antioxidant potential (BAP) or derived from reactive oxygen metabolites (dROM). The rapid measurement of oxidative stress levels in the body has been possible due to point of care (POC) systems, with only a small sample of blood. It is not strange that in a few more years new tools will emerge to diagnose earlier redox imbalance and its consequences in septic patients [62,63] (12)

## Multiple organ dysfunction syndrome

The progression of severe sepsis, septic shock, endothelial dysfunction, and mitochondrial dysfunction, alters the functioning of cells and tissues, ultimately resulting in Multiple Organ Dysfunction Syndrome (MODS), which has been defined as a homeostatic alteration of 2 or more organs that can be reversed through intensive care [64]. The increased levels of ROS, NO and ONOO− in plasma, are involved in the pathogenesis of MODS [65]. Many organs can be affected, such as heart, lungs, liver, gastrointestinal duct, nervous system and kidneys [66].

### Organs diseases

#### Heart diseases

The most common postoperative complications after cardiac surgery include a number of diseases, such as atrial fibrillation and myocardial reperfusion injury. These diseases represent high risks of morbidity and mortality [67]. The immune system plays an important role after being the main mediator of pro-inflammatory and anti-inflammatory cytokines in the different organs. In this way, IL-6 has cardiovascular modulation functions by promoting left ventricular remodeling, inducing systolic dysfunction and altering the activation and response mechanisms in beta-adrenergic receptors on cardiomyocytes. Further, it is also related to effects on myocardial stunning and as a potent negative inotrope [68].

One of the most important complications after heart surgery is the postoperative atrial fibrillation (POAF), that occurs in up to 60% of patients [68]. NADPH oxidase system is associated with the pathogenesis of POAF by the main atrial remodeling. This family is the main source of ROS in cardiomyocytes and is associated with pathologies such as paroxysmal and chronic AF. Likewise, it has been postulated that NOS can also contribute to oxidative stress, because ROS can cause their uncoupling and generating O_2_^·^− instead of NO [69]. Different studies using right atrial appendage samples indicate that NADPH oxidase activity is the most important predictor of developing POAF [70]. Also, the development of POAF in discharge patients is associated with a higher postoperative leukocytes count [71].

Another acute event that is intimately associated with oxidative stress is the acute myocardial infarction. Currently, the gold standard for treating acute myocardial infarction is the restoration of coronary flow through percutaneous primary coronary intervention (PPCI). However, during the process of restoring blood flow, myocardial reperfusion injuries can be paradoxically induced, which accounts for up to 50% of the final size of the infarction. Myocardial reperfusion, that is restoration of blood supply to myocardial tissue after a period of prolonged ischemia, produces local inflammation and increased formation of ROS [58]. Despite PPCI being the gold standard as a treatment, it has irreversible negative outcomes associated with oxidative stress.

Ischemia–reperfusion damage and the pro-oxidant environment are capable of generating pathophysiological ventricular remodeling, with consequent heart failure. Specifically, ROS are capable of altering the electrophysiology and contractile machinery of cardiomyocytes, by modifying proteins such as the sodium-calcium exchanger, sodium channels, potassium channels and L-type calcium channels, which are fundamental for physiology excitatory heart system. ROS also have a negative inotropic capacity, which can alter myocardial contractility and lead to ejection dysfunction of the heart. This is explained because they can alter the activity of the sarcoplasmic reticulum Ca^2+^-adenosine triphosphatase (SERCA), as well as reduce the calcium sensitivity of myofilaments in the contractile syncytium. Another pathological effect of ROS is that it produces an alteration in the energy metabolism of the heart, because it damages the proteins associated with this mechanism. Finally, ROS is capable of activating and promoting the proliferation of cardiac fibroblasts, as well as activating metalloproteinases capable of degrading and remodeling the extracellular matrix of cardiomyocytes, which leads to fibrosis and accentuates heart failure [72]. Furthermore, NADPH oxidase, under conditions of oxidative stress, is capable of increasing ROS production not only due to this pro-oxidant state but also due to different pathological processes such as mechanical stretching, as well as intermediates such as angiotensin II, endothelin-1 and tumor necrosis factor (TNF-α) [73].

#### Liver diseases

The liver is an organ with an exceptional potential to regenerate after resection. About 30% of the future liver remnant is enough to maintain the numerous functions in the body, such as protein synthesis, hormone secretion and detoxification. Thereby, liver surgery supposes a treatment with exceptional results, but in a surgical context, IRI has been demonstrated to cause the production of ROS after reoxygenation of the liver, leading to lipid peroxidation and hepatocellular injury [74]. In some cases, more than 10% of patients can develop a classic complication called postoperative liver failure (POLF) after large resections that might become a major problem because of the association between POLF and other inflammatory complications [75]. Some factors that can act as pathogenic causes of POLF are extensive blood loss, major magnitude of surgery, liver damage status (steatosis, ﬁbrosis or cirrhosis) and postoperative infections [76].

#### Kidney diseases

The kidney is a very sensitive organ to the hypoxic environment. The tubular cells of the thick ascending branch of the renal medulla have the highest oxygen extraction capacity of any cell in the human body, but they are also considered to be one of the most sensitive to sudden changes in oxygen levels [77]. The fact that Na is reabsorbed in this area is that it also needs high levels of ATP, which are provided by the respiratory chain in the mitochondria, being in direct relationship with the levels of O_2_ in the blood [78]. A severe and common complication after a cardiac surgery is acute kidney injury (AKI), with an occurrence up to 30% of patients [79]. Kidney receives about 25% of total blood supply, so in a state of oxidative stress is susceptible to damage from ROS and from metabolic products that act as inflammatory ‘toxins’, leading to AKI and hemodynamic imbalance [78]. After reperfusion, the injury expands and is distinguished by an inflammatory response that progresses to oxidative stress [80]. The progression of AKI is generally divided into the phases of initiation, extension, maintenance, and recovery. Especially in the first phase, an ischemic process occurs, usually abrupt, which leads to parenchymal cells not being perfused correctly and causing different mechanisms of cellular injury and their subsequent death, either by apoptosis or necrosis [78]. The purpose of this review is not to delve into this topic, especially due to its depth and complexity. However, it is important to consider some specific new biomarkers that have started to be used in different clinical criteria today to identify an AKI in the shortest possible time. Some of these biomarkers are, for example, urinary α1-microglobulin, π-glutathione transferase, and serum and urine neutrophil gelatinase-associated lipocalin (NGAL) [81].

There comes a time such that the damage is already irreversible and progressive, which causes invasive therapies to be established as the only solution. Renal transplantation is considered the ‘gold standard’ treatment for end-stage renal disease (ESRD), but just like PPCI treatment in acute myocardial infarction, this intervention can be related with oxidative stress, with irreversible consequences [82]. There are many sources of ROS production, being IRI the most associated with oxidative stress [30]. In the renal cells, IRI causes impairment in cytoskeletal integrity and changes in epithelial cell polarity, producing loss of proximal tubule brush border, inflammation, microvascular dysfunction, necrosis and apoptosis [83]. As a result of cell damage, reperfusion is capable of aggravating the inflammatory condition because it is capable of attracting neutrophils, which are capable of infiltrating kidney cells and causing greater damage, and can also trigger long-term fibrosis of the renal parenchyma [84].

## Antioxidant therapy

The use of antioxidant therapy in states of oxidative stress damage is well known. These dysfunctions may originate in the mitochondrial transport chain, which is classified as the most important in the pathogenesis of sepsis. There are multiple types of antioxidants, which act on different targets and with different clinical efficacy [44]. The role of the antioxidants most used in clinical trials and those of greater knowledge in their mechanism of action will be briefly described.

- **Vitamin C:** As known as ascorbic acid, is an essential antioxidant capable of donating electrons to both enzymatic and non-enzymatic processes. Its use as a cofactor is to get involved in processes such as collagen synthesis, preventing lethal genetic mutations, protecting leukocytes and helping in the production of carnitine, related to metabolic energy [85]. Ascorbic acid is capable of reacting with the oxidized tocopheroxyl radical bound to the membrane, generating a reduction and producing active tocopherol, which is this compound that has antioxidant functions [86]. Vitamin C has been shown to be capable of attenuating the intracellular increases of iNOS mRNA, as well as the expression of COX and TNF-a mRNA in cells of the immune system. So, a regulatory role has also been seen in the gene expression of pro-oxidant and pro-inflammatory molecules with the use of ascorbic acid [87].
- **Vitamin E:** Vitamin E is a fat-soluble antioxidant, where its main active compound is alpha-tocopherol. There are various studies on the efficacy of vitamin E as an antioxidant potential in different pathologies. Alpha-tocopherol prevents lipid peroxidation of cell membranes, as well as inhibiting the synthesis of free radicals by forming a low-density derivative, which is unable to attack the lipid components, such as the membrane, of the cell [88]. Alpha-tocopherol has been shown to be able to positively alter oxidative stress biomarkers, such as SOD, catalase, etc. [89]. The use of this antioxidant has been evaluated with its use in high doses, resulting in an inhibition of proatherosclerotic processes in endothelial cells. Its mechanism of action in this process is through the reduction of superoxide radical anions and IL-1β by activated monocytes, lipid peroxidation, endothelial adhesion of monocytes and platelet aggregation [90].
- **N-acetylcysteine:** In septic conditions, in recent years an important therapeutic utility has been seen with the use of N-acetylcysteine (NAC). This treatment produced a decrease in organ damage biomarkers (Cre, CPK, ALT and AST) and inflammatory biomarkers (TNF-a, IL-6 and IL-10) caused by sepsis [91]. Multiple studies have proposed that NAC has a hepatoprotective effect by attenuating mediators of oxidative stress and inflammation, as well as an improvement of the same parameters in metabolic syndrome [92]. Another study, in cases of respiratory distress, demonstrated a dose-dependent decrease in lung NF-κB and a lower expression of chemokine mRNA in lung tissue. Thus, NAC appears to have a role in regulating the pulmonary inflammatory response through the activation of NF-κB [93].
- **Selenium:** This element is vital for the functioning of endogenous antioxidant enzymes, such as GSH-Px and thioredoxins. Low levels of selenium could affect the correct oxidative balance in the body [94]. At the cellular level, selenium deficiency produced an increase in the expression of iNOS in macrophages, which increased the synthesis of NF-kB and its consequent oxidative damage at the systemic level. An inverse relationship has been observed between cellular selenium levels and iNOS expression in macrophages stimulated by LPS [95].

## Novel treatment of antioxidants in POAF

For the treatment of POAF it has been postulated that the use of antioxidants is able to decrease the development of inflammation and oxidative stress that occurs in the heart. In a randomized controlled trial made by Rodrigo et al. [96] showed that the combined use of a triple therapy with vitamin E, vitamin C and of n-3 polyunsaturated fatty acids (n-3 PUFAs) favorably affected postoperative atrial fibrillation, increased antioxidant potential, and attenuated oxidative stress and inflammation. Specifically, n-3 polyunsaturated fatty acids can act as indirect antioxidants by inducing low to moderate increases in ROS levels and decreasing vulnerability of myocardial tissue to a subsequent oxidative challenge. With this, it is proposed and reaffirmed that the use of this associated tri-therapy is a cost-effective long-term strategy to prevent and reduce oxidative stress damage in myocardial cells, and thus avoid POAF and the health and monetary costs involved.

## Conclusions

Already being mentioned the pathophysiology of oxidative stress, its knowledge becomes imperative. It has been shown that oxidative stress is a common factor in many pathophysiological mechanisms, such as sepsis, inflammation and even organ diseases. ROS/RNS increase the damage in processes such as sepsis, being able to produce a mitochondrial decoupling that viciously increases the injury due to oxidative stress and sepsis. Also, there are diseases that are based on oxidative stress damage, such as acute myocardial infarction or AKI, so it is necessary to consider oxidative levels in these pathologies to bring the best therapy with the least possible side effects. In this way, a variety of possible treatments can be used to counteract the possible damages produced by the excess of ROS and RNS in the human body. One of the most attractive alternatives is the use of exogenous antioxidants, such as vitamin C and E, which in many clinical trials have been catalogued as a solution to the irreversible damage that oxidative stress can cause. The joint action of multiple antioxidants can produce a protective effect against oxidative stress damage.
